# Aplasia Cutis Congenita in Bart's Syndrome: A Case Report and Literature Review

**DOI:** 10.1002/ccr3.72378

**Published:** 2026-04-03

**Authors:** Parvaneh Sadeghimoghadam, Niloofar Hoorshad, Maryam Ghavami Adel, Mahroo Rezaeinejad, Marjan Ghaemi

**Affiliations:** ^1^ Breastfeeding Research Center, Family Health Research Institute Tehran University of Medical Sciences Tehran Iran; ^2^ Department of Obstetrics and Gynecology, Imam Khomeini Hospital Complex, School of Medicine Tehran University of Medical Sciences Tehran Iran; ^3^ Vali‐E‐Asr Reproductive Health Research Center, Family Health Research Institute Tehran University of Medical Sciences Tehran Iran

**Keywords:** Aplasia cutis congenita, Bart's syndrome, congenital diseases, congenital skin disorder, neonatal mortality

## Abstract

Aplasia cutis congenita (ACC) is a partial or complete absence of skin layers which can be a part of a syndromic disease. Bart's syndrome is a combination of clinical manifestations including ACC, epidermolysis bullosa, blistering in the oral mucosa, nail dystrophy, or congenital absence of nails. Additional anomalies, such as pyloric atresia, a broad nasal root, hypertelorism, and ear abnormalities, may also be present. We aimed to raise awareness about concomitant anomalies with ACC. A 23‐year‐old female with gravid 3 para 2 gave birth to a male fetus of 36 weeks and 3 days who had multiple skin lesions on bilateral temporal skin of scalp, left buccal, and dorsum of left hand. Small white‐colored pustules were prominent on his chest, and nail dystrophy on the first finger of the right hand was reported. Both ears were anomalous and right‐sided torticollis was observed. There was no bony defect at the site of the skin lesions. On the third day of admission, duodenal atresia was diagnosed owing to vomiting and abdominal distention and the presence of double bubble sign on abdominal x‐ray. The patient underwent surgery on the fifth day of birth and remained intubated until the 25th day of birth and unfortunately passed away. The management of ACC alone can be either surgical or conservative; however, it is important to first rule out concomitant anomalies and syndromes which include ACC as a cutaneous manifestation.

## Key Clinical Message

The management of aplasia cutis congenita (ACC) in Bart's syndrome alone can be either surgical or conservative; however, it is important to first rule out concomitant anomalies and syndromes, which include ACC as a cutaneous manifestation.

## 1 Introduction

Aplasia cutis congenita (ACC) is a congenital cutaneous disorder that is defined by absence of skin layers to varying degree, which can be either localized or widespread [1]. Its incidence is nearly 1–3 cases out of every 2000–10,000 live births, and there is no gender predilection reported. Although the precise pathophysiology of ACC is yet to be identified, two most probable mechanisms contributing to ACC are: (a) disruption or failure in the development of skin layers, including epidermis, dermis, and subcutaneous fat, or (b) in utero destruction of normally developed skin [2].

Skin lesions can occur on almost any part of the body; however, more than 80% of ACC cases involve the scalp, particularly the vertex. The presentation of lesions accordingly consists of superficial erosions to deep ulcers, which are usually covered with a thin, transparent membrane. These membranous lesions may be isolated or present as a group of scattered defects [3, 4]. On the other hand, cases characterized by large, irregular, or stellate scarring defects are referred to as non‐membranous or classic aplasia cutis. This form is frequently associated with underlying bone defects, which may expose the dura, sagittal sinus, or even the brain [5].

The main differential diagnosis of ACC includes encephalocele, dermoid cyst, neonatal herpes, focal dermal hypoplasia, amniotic band syndrome, and nevus sebaceous [6]. To distinguish ACC from these conditions, it requires thorough clinical information, including the patient's demographics, family and obstetric histories, as well as a detailed examination of the skin lesion. The properties of the lesion such as the number, location, size, shape, the presence of a hair collar sign and radiologic findings play a crucial role in accurate diagnosis [3].

ACC can be a component of several syndromes including Adams‐Oliver syndrome, Bart's syndrome, Carmi syndrome, Oculo‐Ectodermal syndrome, Wolf‐Hirschhorn syndrome, trisomy 13, Goltz syndrome, Ellis‐van Creveld syndrome, Setleis syndrome, and Patau syndrome. These syndromes may involve multiple organs, including the skin, eyes, ears, nose, extremities, as well as the cardiovascular, gastrointestinal, genitourinary, and central nervous systems [7, 8, 9].

Here, we present a case of Bart syndrome with ACC, which unfortunately resulted in the patient's death at 25 days of age.

## 2 Case History/Examination

A 23‐year‐old female gravid 3 para 2 living child 2 with gestational age of 36 weeks and 3 days presented to the emergency ward due to labor pain and uterine contractions. She had a history of two natural vaginal deliveries which resulted in two completely healthy boys. Cervical dilation was 4 cm with effacement of 30%. Antepartum anomaly scan and screening tests were reported normal. Her family and past medical history were unremarkable and no consanguinity was mentioned.

The mother had been taking only antenatal multivitamins and ferrous sulfate. She was admitted to the obstetrics ward for vaginal delivery, but because of repetitive late fetal heart rate decelerations, emergent cesarean section was planned.

## 3 Differential Diagnosis, Investigations and Treatment

**FIGURE 1:**
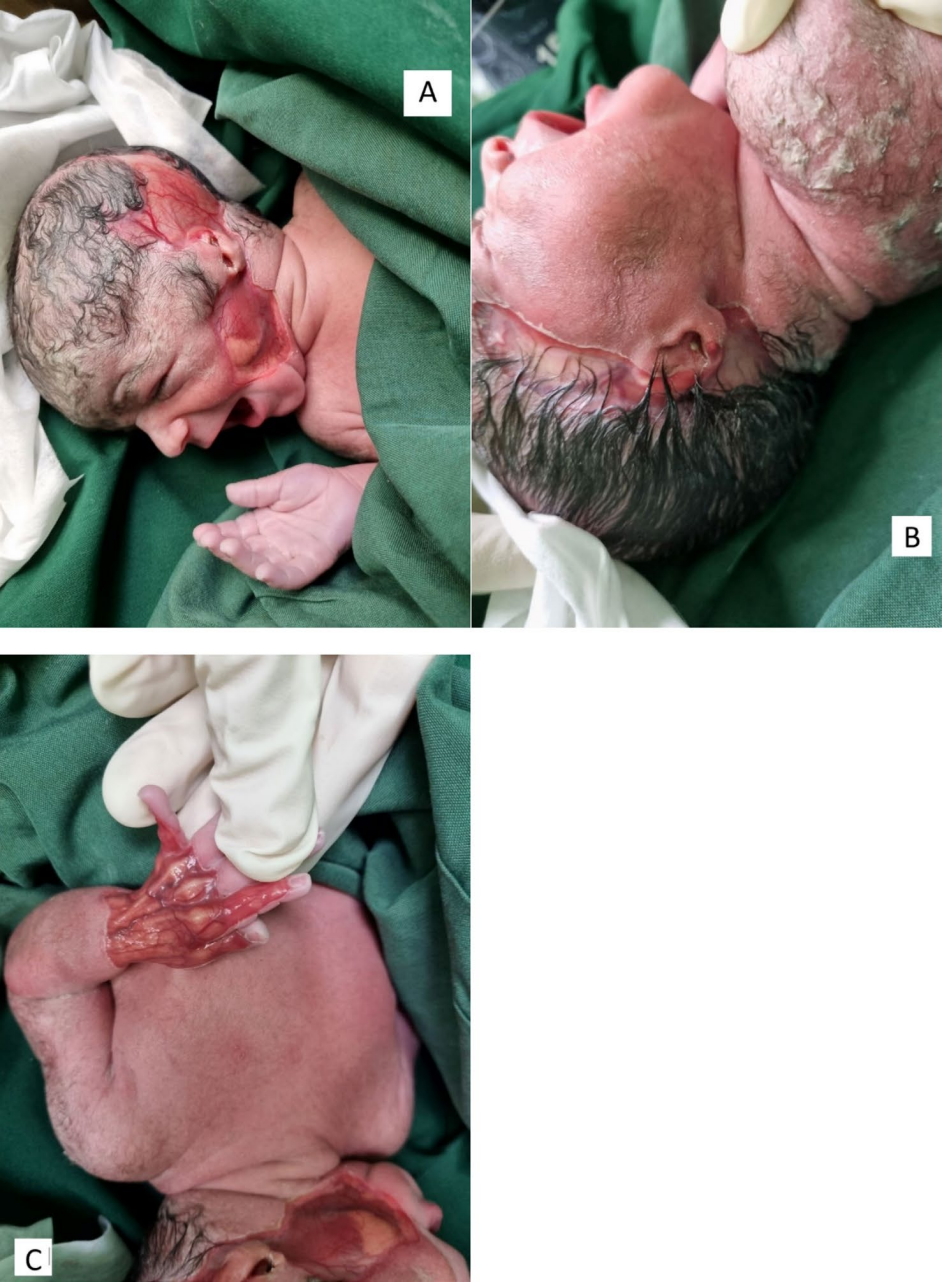
Clinical photographs of the lesion. (A) Right temporal skin of scalp measuring 6X4 cm and left buccal lesion measuring 3X3 cm, (B) Left temporal skin of scalp measuring 7X2 cm, (C) Dorsum of left hand measuring 4X3 cm.

In operating room, obstetric surgeon reported polyhydramnios and a baby boy with APGAR score 9/10 was born. On examination, multiple skin lesions on bilateral temporal skin of scalp (right: 6X4 cm, left: 7X2 cm), left buccal (3X3 cm) and dorsum of left hand (4X3 cm) were notable Figure 1.

Small white‐colored pustules were prominent on his chest and the mother denied any viral infection like varicella zoster or contact with an infected person during her pregnancy period. Also, nail dystrophy on the first finger of the right hand was reported. Both ears were anomalous and right‐sided torticollis was observed. There was no bony defect at the site of skin lesions. The baby weighed 2590 g with a height of 45 cm. Regarding prematurity and aplasia cutis, he was admitted to the neonatal intensive care unit.

## 4 Conclusion and Results (Outcome and Follow‐Up)

Routine care including supplementary oxygen, appropriate hydration, antibiotics, dressing of lesions with normal saline soaked gauze and then a regimen of topical gentamycin was implemented. Dermatology, cardiology, ophthalmology consult and brain ultrasound were requested. Echocardiography and brain sonography were normal. On the third day of admission, duodenal atresia was diagnosed owing to vomiting and abdominal distention and the presence of double bubble sign on abdominal x‐ray.

Patient underwent surgery on the fifth day of birth, and due to atretic D1, duodenostomy was performed. After one week of hospitalization, metabolic acidosis and electrolyte abnormalities made his condition poor. Chest x‐ray revealed upper lobe collapse in the right lung. Respiratory physiotherapy was ordered and Non‐Invasive Positive Pressure Ventilation (NIPPV) administered. On the 13‐day‐old, the baby was intubated and electrolyte imbalance was exacerbated. On the 16‐day‐old, the patient had generalized tonic clonic seizure and phenobarbital was given. He remained intubated until the 25th day of birth and unfortunately expired. Placental pathology report indicated that the placental membrane had meconium‐laden macrophages and subchorionitis. Placental villi showed accelerated villous maturation which is compatible with maternal vascular malperfusion. Umbilical cord had normal pathology report. Parents refused to send the deceased baby for autopsy, and they never returned for post‐partum visits.

## 5 Discussion

ACC is a rare condition, and its exact pathogenesis remains incompletely understood. Since 70%–90% of lesions are located on the scalp, it has been hypothesized that tension‐induced disruption of the overlying skin occurs between 10 and 15 weeks of gestation, coinciding with the period of hair patterning, directional growth, and rapid brain expansion. This hypothesis also offers an explanation why scalp ACC often occurs near the hair whorl, a region thought to experience the greatest tensile stress during rapid brain growth [6].

ACC has a highly variable presentation and its diagnosis is clinical. On physical examination, the most common presentation of ACC is ulcerations or erosions of the skin, which may extend into underlying structures such as muscle or bone, in 15%–20% of ACC cases [1]. Ultrasound study can have a substantial role in the diagnosis of bone defects [4]. In some instances that start to heal while the fetus is unborn, ACC may present simply as an atrophic scar at birth [3].

Prenatal application of ultrasound was first proposed by Meizner and Carmi et al. [10], in 1990 and they described a new ultrasound “snowflake” sign to establish diagnosis of intrauterine skin‐exfoliation syndrome. Generally, the skin of unborn fetus in ultrasound study shows strong echoes and in cases of ACC, these strong echoes will vanish. Recently, 3D reconstruction of ultrasound imaging has been used more frequently to assess morphology of fetal organs specifically extremities and can provide the detailed morphology of fingers and toes. Fetus with ACC shows localized disruption of skin continuity on 3D ultrasonography. Conventionally, surface‐mode imaging is used to recognize surface anatomy, integrity of skin cover, and topographic relationships between the segments of each limb [11].

Classifying ACC has proven challenging due to the unclear etiology of many cases. Earlier studies tried to categorize ACC based on associated abnormalities, inheritance patterns, and affected body regions, but recent advances in DNA sequencing techniques have shown promise as a more accurate classification method. However, the high cost and limited availability of genetic sequencing have restricted its widespread application. In this regard, Humphrey et al. proposed a practical classification approach based on the location and shape of the lesion, which helps clinicians identify associated findings and manage the potential anomalies accompanying the ACC [2].

In our case, the combination of scalp and upper extremity ACC, nail dystrophy on the first finger of the right hand, duodenal atresia, and blistering lesions resembling epidermolysis bullosa (EB) suggested Bart syndrome as a potential diagnosis.

Bart's syndrome is defined by the coexistence of ACC and EB and was first described in a family with multiple affected members. Common features include ACC, which may affect not only the scalp but also the trunk and extremities. Other findings often associated with Bart's syndrome include blistering in the oral mucosa, nail dystrophy, or congenital absence of nails. Additional anomalies, such as pyloric atresia, a broad nasal root, hypertelorism, and ear abnormalities, may also be present [12]. Table 1 summarizes characteristics of most recently reported cases of Bart's syndrome.

**TABLE 1:** Characteristics of most recently reported cases of Barts Syndrome.

First author	Gestational age	Skin lesion localization	Accompanying anomalies	Systemic examination	Risk factor	Skin biopsy	Parental consanguinity	Management
Alfayez [13]	Term	Anteromedial aspect of both lower legs	Dystrophic nails of some fingers	Normal	No	Subepidermal blister	No	Conservative
Kulali [14]	Term	Anteromedial aspect of both lower legs	Blisters on the upper lip mucous membranes and on the left wrist	Normal	No	Subepithelial vesicle formation	No	Conservative
Gang [15]	Term	Right knee to dorsum of ipsilateral foot	Flaccid and tense bullous lesions on the face, oral mucosa, upper limbs and back containing translucent fluid	Normal	Not mentioned	Subepidermal cleft	Not mentioned	Conservative
Alharthi [16]	38 weeks + 5 days	Whole left leg and the lower part of the left thigh	No	Low birth weight	History of epidermolysis bullosa in 2 siblings	Confirmed epidermolysis bullosa	First‐degree relative	Conservative
Sharifi [17]	36 weeks + 4 days	Anteromedial part of the shin, the anterolateral aspect of the neck, periauricular, nose and both hands	Blisters over the previous lesions. Blistering over normal skin in response to minor trauma or friction	No	No	Subepidermal blister	No	Conservative
McGowan [18]	Term	Circumferential bilateral lower extremity ulcerations and skin hypoplasia over the same region	Dystrophy of several toe nails involved with the ulcerations	Not mentioned	In utero exposure to cocaine and alcohol	Central area of thin epidermis with underlying elastolysis, fibrosis, and an absence of adnexal structures	Not mentioned	Conservative
Eghtedari [19]	33 weeks	Absence of skin formation in head, face, neck, left hand and genitalia	Left ear lobe aplasia and absent right ear, corneal cloudiness, white appearance of the pupils, bilateral cryptorchidism	No	No	Not mentioned	Closely related	Conservative
Shahidi‐dadras [20]	Not mentioned	Absence of skin on the anteromedial aspect of the right knee, leg, and foot	Bilateral clubfoot and skeletal deformity of the right tibia and fibula	No	No	Subepidermal blister and scattered polymorphonuclear cells	No	Conservative
Han [21]	37 weeks	Anteromedial aspect of both lower extremities	Lesions in the oral cavity	No	No	Subepidermal blister formation	No	Conservative

The genetic defect traditionally associated with Bart's syndrome involves collagen VII, which disrupts the interaction of anchoring fibrils at the dermal‐epidermal junction [22]. Diagnostic work‐up for Bart's syndrome includes a skin biopsy to evaluate for genetic mutations and immune mapping to determine the EB subtype. If pyloric atresia is suspected, further imaging is warranted.

Carmi Syndrome is an exceptionally rare autosomal recessive genetic disorder that closely resembles Bart's syndrome. It is characterized by a triad of pyloric atresia, junctional EB, ACC in approximately 28% of cases. Bart's syndrome is primarily diagnosed based on the presence of ACC and EB. As noted earlier, it may also involve nail abnormalities, such as congenital absence or nail dystrophy, though these are not essential for diagnosis. Studies have reported that nearly 30% of Bart's syndrome cases include extracutaneous abnormalities, with pyloric atresia and ear malformations being the most common. Furthermore, a systematic review found that Bart's syndrome occurred in 28% of patients with Carmi Syndrome, highlighting the challenges in distinguishing between these two rare conditions [13].

Different treatment approaches for ACC have been documented, including the use of petrolatum, bacitracin, occlusive dressings, wet‐to‐dry dressings or saline drips, betadine, silver sulfadiazine, and biological or synthetic skin substitutes. The average healing time for conservatively managed cases is approximately 27.9 days. Conservative treatment is typically preferred for both small and large lesions located on the trunk [7].

Systemic antibiotics are frequently administered to patients, particularly those with scalp lesions, until wound healing is complete to prevent severe infections, including meningitis [19]. Ancillary treatments such as physical and occupational therapy may also be necessary, particularly for extremity lesions, to prevent complications such as flexion contractures [23].

Potential complications of conservative treatment include infection (such as meningitis), bleeding due to desiccation (particularly dangerous over the sagittal sinus), and prolonged healing times. Once the wounds heal, residual issues such as hypertrophic scars, atrophic plaques, or cicatricial alopecia may persist [9].

Surgical management options for ACC include excision and closure, skin grafting, local flaps, and tissue expansion. While some authors prefer emergent surgery shortly after birth, others recommend waiting several days to weeks before intervening. Although conservative treatment is often the initial approach, early surgical intervention for large midline scalp lesions may reduce complications and improve survival outcomes [24]. For midline lesions, preoperative imaging with computed tomography (CT) or magnetic resonance imaging (MRI) is essential to evaluate underlying bony defects or potential intracranial connections [8, 25].

In conclusion, whenever a case of ACC is suspected, it's important to consider concomitant anomalies and associated syndromes before initiating treatment of skin defects. Complete wound coverage can be accomplished through conservative management, surgical intervention, or a combination of both. While complications can occur with either approach, outcomes are generally favorable.

## Author Contributions

**Parvaneh Sadeghimoghadam:** conceptualization. **Niloofar Hoorshad:** writing – original draft, writing – review and editing. **Mahroo Rezaeinejad:** investigation. **Maryam Ghavami Adel:** writing – review and editing. **Marjan Ghaemi:** supervision.

## Funding

The authors have nothing to report.

## Consent

Written informed consent was obtained from the parents or legal guardians of all participating children. This study was approved by the Tehran University of Medical Sciences Human Research Ethics Committee.

## Conflicts of Interest

The authors declare no conflicts of interest.

## Data Availability

The data of the case is available upon request.
